# Contact tracing: Unearthing key epidemiological features of COVID-19

**DOI:** 10.1177/2050313X20933483

**Published:** 2020-06-24

**Authors:** Pranav Ish, Sumita Agrawal, Akhil Dhanesh Goel, Nitesh Gupta

**Affiliations:** 1 Pulmonary Medicine, Safdarjung Hospital, New Delhi, India; 2 Pulmonary medicine, Medipulse Hospital, Jodhpur, India; 3 Community Medicine & Family Medicine, All India Institute of Medical Sciences, Jodhpur, India; 4 Safdarjung Hospital, New Delhi, India; 5 Department of Pulmonary, Critical Care and Sleep Medicine, VMMC and Safdarjung Hospital, New Delhi, India

**Keywords:** COVID-19, contact tracing, prevention, screening

## Abstract

COVID-19 is an emerging global pandemic with a steady rise in both morbidity and mortality over the past few months. Contact tracing of COVID-19 patients is an essential task to mitigate the spread. The following case was one of the initial patients reported from India and details the importance of contact tracing, timely testing and adequate quarantine/isolation in disease control.

## Introduction

Contact tracing of a confirmed case is the first community-based step recommended by the World Health Organization (WHO) for rapid detection of COVID-19 clusters and promptly reduce human-to-human transmission, thereby preventing outbreaks and delaying the spread of disease.^1^ The WHO has documented that pre-symptomatic transmission of COVID-19 can occur in the incubation period which is generally 5–6 days but can last up to 14 days.^2–8^ Asymptomatic transmission, on the contrary, refers to transmission from a person who never develops symptoms. Even though the WHO has documented asymptomatic cases through screening and contact tracing, asymptomatic transmission has not yet been reported.^9^ However, due to its respiratory and direct spread mechanisms, introduction of a single case to a new region has the potential to create exponential clusters if not controlled on time. An interesting study from Singapore of over 240 cases of COVID-19 over nearly 2 months identified seven cluster of cases with pre-symptomatic transmission being the most likely cause of spread.^6^ We share an initial experience of contact tracing of one of the initial few cases from a tertiary care centre from India.

## Case

The index case, a 44-year-old gentleman, who had been on a business trip to Italy for 12 days, returned to New Delhi on 25 February. He alighted a flight of more than a hundred passengers from various countries. At the international airport screening, he was found to be asymptomatic and was advised home quarantine. On 26 February, ignoring his quarantine instructions, the apparently fit man travelled by bus from Delhi to Agra, Uttar Pradesh – around 230 kms away. There were about 40 other passengers in the same bus. The index case reached his home and met his aged parents (73 and 64 years old), his wife (37 years) and son (16 years). The next day, he invited his close friend and his wife for dinner. During this whole time, he felt he was a healthy man.

On 1 March, he noticed a mild cough and fever. When a visit to the local practitioner did not relieve his symptoms, he approached a tertiary care centre a day later where he was rightfully suspected as a case of COVID-19 and referred to our hospital. The transportation back from Agra to New Delhi was in a dedicated ambulance after wearing full personal protective equipment (PPE). At our hospital, he was placed in an isolation room at the designated COVID-19 facility. His nasopharyngeal and oropharyngeal swabs were taken and sent for COVID-19 testing by polymerase chain reaction (PCR)-based test, the only approved test at the time. He was confirmed positive on 3 March.

The contact tracing was immediately initiated. Subsequently, his parents, wife and son also tested positive. Three days later, his close friend and his wife, with whom he had dined not a week back, both tested positive. All seven cases, three of them symptomatic, stayed in isolation ward of our hospital. The timeline is summarized in Table 1.

**Table 1.** The timeline of exposures to our asymptomatic index case.

	February						March																
	25	26	27	28	29	1	2	3	4	5	6	7	8	9	10	11	12	13	14	15	16	17	18
Index case	Flight^a^	Bus^b^						+			+			+		+						–	–
Wife								+						–		–							
																							
Son								+						+		+						–	–
Father								+			–			–		–							
Mother								+			–			–		–							
Friend											+										–	–	
Friend’s wife											+										–	–	

a Flight from Italy to India

b Bus from New Delhi to Agra.

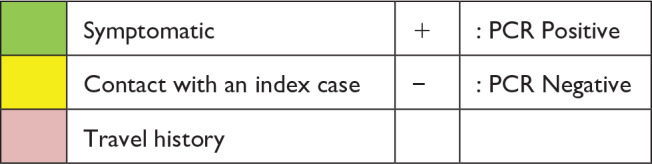

One hundred thirty-six contacts of this index case were traced and tested for COVID-19, all of them were negative, including the housemaid and her family, bus passengers, neighbours and the local doctor consulted and patients in the clinic at that time.

All seven patients admitted, two of them elderly, recovered completely. However, even on the 14th day since return, the index case continued to be PCR positive along with his son. They were kept admitted till two nasopharyngeal swabs were reported negative for COVID-19 as per national policy.^10^ The other family members had turned PCR negative and were discharged accordingly.

## Discussion

This case highlights the infectivity of the virus through pre-symptomatic people. COVID-19 has globally impacted social and economic life, but the loss of life is irreplaceable and irreparable. A certain set of precautions and vigilance including hand hygiene, PPE, avoiding unnecessary travel, quarantine and self-isolation are the most critical steps in containing this pandemic illness from getting out of control.

India is a nation of 1.3 billion people, densely populated, and resource constrained. If we face an outbreak of the same ferocity as China, Italy or Spain, our healthcare resources would be overrun a lot sooner. The mere thought of the consequences is petrifying. Our only saving grace – we are probably in the initial phases of the epidemic.^10,11^ Until this stage, the authorities are largely able to keep track and quarantine the cases and their contacts. But beyond this, it becomes impossible. This is our final defence, our ultimate stand. Any further, and we may reach a tipping point of our reserves. Healthcare and economic collapse would be inevitable. So, the buck stops with strict self-isolation and quarantine.

The screening of passengers at International airports in India was started from 18 January 2020^12^ and more than 1.5 million passengers have since arrived in India.^13^ The Ministry of Health and Family Welfare along with state health authorities has made a detailed list of these passengers and have embarked on an extensive tracking of these passengers to ensure quarantine and isolation as per incubation-period recommendations.^14^ The adequacy of contact tracing will be crucial determinant towards the control of this corona virus disease. However, due to high proportion of pre-symptomatic having potential to promulgate secondary and tertiary transmissions, timely contact tracing may not be feasible especially due to the known delays of reporting and laboratory test results. Thus, contact tracing may only have a supplementary role to other restrictive strategies like social distancing and avoidance of unnecessary social contact through administrative measures like lockdown.

## Conclusion

Understanding the disease epidemiology is a first step in response preparedness. COVID-19 has a risk of unprecedented spread especially by means of pre-symptomatic transmission. Contact tracing, timely testing and adequate quarantine/isolation will go a long way in reigning the rapid spread of this disease.
